# Methyltransferase like 3 promotes colorectal cancer proliferation by stabilizing CCNE1 mRNA in an m6A‐dependent manner

**DOI:** 10.1111/jcmm.15042

**Published:** 2020-02-10

**Authors:** Wei Zhu, Yan Si, Jun Xu, Yu Lin, Jing‐Zi Wang, Mengda Cao, Shanwen Sun, Qiang Ding, Lingjun Zhu, Ji‐Fu Wei

**Affiliations:** ^1^ Research Division of Clinical Pharmacology The First Affiliated Hospital of Nanjing Medical University (Jiangsu Province Hospital) Nanjing China; ^2^ Department of General Surgery The First Affiliated Hospital of Nanjing Medical University (Jiangsu Province Hospital) Nanjing China; ^3^ Department of Oncology The First Affiliated Hospital of Nanjing Medical University (Jiangsu Province Hospital) Nanjing China; ^4^ Department of Urology The First Affiliated Hospital of Nanjing Medical University (Jiangsu Province Hospital) Nanjing China

**Keywords:** butyrate, CCNE1, colorectal cancer, intestinal microbiota metabolites, m6A, methyltransferase like 3

## Abstract

m6A modification is the most prevalent RNA modification in eukaryotes. As the critical N6‐methyladenosine (m6A) methyltransferase, the roles of methyltransferase like 3 (METTL3) in colorectal cancer (CRC) are controversial. Here, we confirmed that METTL3, a critical m6A methyltransferase, could facilitate CRC progression in vitro and in vivo. Further, we found METTL3 promoted CRC cell proliferation by methylating the m6A site in 3′‐untranslated region (UTR) of CCNE1 mRNA to stabilize it. Moreover, we found butyrate, a classical intestinal microbial metabolite, could down‐regulate the expression of METTL3 and related cyclin E1 to inhibit CRC development. METTL3 promotes CRC proliferation by stabilizing CCNE1 mRNA in an m6A‐dependent manner, representing a promising therapeutic strategy for the treatment of CRC.

## 1 INTRODUCTION

Colorectal cancer (CRC) is one of the frequent cancers in the world. It remains the second leading cause of cancer‐related deaths with high morbidity.^1^ For early diagnosis and reasonable treatment of CRC, the 5‐year survival rate can reach to 90%.^2^ However, for CRC patients with advanced metastases, the 5‐year survival even reduces to 8%.^2^ Therefore, it is essential to understand how CRC develops and find vital therapeutic targets for the treatment of CRC.

The growing evidence suggests that the development of CRC is a complex process, which involves the changes in the levels of genetics, epigenetics and transcriptomics.^3^, ^4^, ^5^ mRNA modifications represent another layer of epigenetic regulation of gene expression, which has been termed ‘RNA epigenetics’ or ‘epitranscriptomics’. Among these, m6A modification is the most prevalent one in eukaryotes,^6^ which occurs in mammalian mRNAs,^7^, ^8^ long noncoding RNAs,^9^ micro RNAs,^10^, ^11^ involving in many RNA functions such as mRNA stability,^7^ splicing,^12^ transport,^13^ localization and translation,^8^, ^14^ primary microRNA processing 10 and RNA‐protein interactions.^15^ The levels of m6A modification in mRNAs were usually controlled by so‐called ‘reader’, ‘writer’ and ‘eraser’.^16^, ^17^ The ‘writer’, which is a high conserved methylase complex, containing methyltransferase like 3 (METTL3),^18^ methyltransferase like 14,^18^ Wilms’ tumour 1‐associating protein,^19^ RNA‐binding motif protein 15,^20^, ^21^ Vir like m6A methyltransferase associated,^18^, ^22^ Fl(2)d‐associated complex component 20 and Hakai.^23^

Recent studies showed that m6A modification played essential roles in tissue development, stem cell formation and differentiation,^24^, ^25^ control of heat shock response 26 and circadian clock controlling,^27^ as well as in tumours formation.^28^, ^29^, ^30^, ^31^, ^32^, ^33^, ^34^, ^35^ As the critical m6A methyltransferase, the role of METTL3 in tumours is controversial. Some studies reported that METTL3 played an oncogenic role in myeloid leukaemia,^29^ liver cancer,^28^ breast cancer,^30^ glioblastoma,^31^ bladder cancer 11 and lung cancer.^33^ Other studies indicated that METTL3 played a tumour suppressor in renal cell carcinoma 34 and glioblastoma.^35^ These studies suggested the effect of METTL3 on tumour development may be tumour‐specific. Abnormal cell proliferation is the main characteristic in cancer cells, which damage the genes that directly regulate their cell cycles. Carcinogenesis made their greatest effect by targeting the regulators of G1 phase progression.^36^ It was found that METTL3 could participate in tumour growth and progression by regulating the cell cycle of cancer cells. For example, METTL3 was identified as an essential oncogene to promote cell cycles and growth in acute myeloid leukaemia by targeting transcription factor SP1.^29^ METTL3 acted as a tumour suppressor in renal cell carcinoma cells to regulate cell cycle arrest in G1 phase by targeting PI3K/Akt/mTOR signalling pathway.^34^

In CRC, a recent research has verified that METTL3 could promote cell self‐renewal, stem cell frequency and migration through an m6A‐IGF2BP2‐dependent mechanism.^37^ However, another study showed that METTL3 could suppress colorectal cancer proliferation and migration through p38/ERK pathways.^38^ Therefore, the role of METTL3 in CRC might be controversial and need to be further explored. In our present study, we confirmed that METTL3 could promote CRC cells proliferation and revealed a new mechanism by directly regulating cyclin proteins in an m6A‐dependent manner.

Human intestinal microbiota can promote or suppress CRC, not only due to the carcinogenic activities of pathogenic bacterium but also caused by the complex effect of wider microbial community, particularly their metabolites, such as deoxycholic acid (DCA), ursodeoxycholic acid and butyrate.^39^ Recent studies demonstrated that microbiomes have strong effects on the m6A modification and METTL3 expression in mouse brain and intestine.^40^ However, whether human intestinal microbiota and their metabolites can affect the CRC m6A modification was still unknown. As a classical intestinal microbial metabolite, butyrate is an abundant (up to >10 mmol/L) short‐chain fatty acid that is transported into the colonic epithelium and localizes within two subcellular compartments.^41^, ^42^ It undergoes β‐oxidation inside the mitochondria and accounts for ≥70% of the energy used by normal colonocytes,^43^ and it also functions as a histone deacetylase (HDAC) inhibitor inside the nucleus to epigenetically regulate gene expression.^44^, ^45^ In a study by Scheppach et al,^46^ human colonic biopsies were exposed to butyrate ex vivo for 4 hours, which revealed that butyrate increased the proliferation rate at the basal 60% area of the crypt.^47^ Butyrate also has the function of resisting inflammation and tumours.^48^, ^49^, ^50^, ^51^, ^52^ In the present study, we found that METTL3 was up‐regulated in CRC tissues and associated with poor survival. It promoted CRC cell proliferation by targeting cyclin E1 by methylating the m6A site in 3′‐untranslated region (UTR) of CCNE1 mRNA. Moreover, we found butyrate could down‐regulate the expression of METTL3 and related cyclin E1, inhibiting CRC development.

## 2 MATERIALS AND METHODS

### 2.1 Clinical specimens and cell lines

Colorectal cancer tissues and their adjacent nontumorous samples were obtained from patients of the First Affiliated Hospital of Nanjing Medical University. All specimens were immediately frozen and stored in liquid nitrogen after surgical removal. The experiments were undertaken with the understanding and written consent of each subject. The study methodologies conformed to the standards set by the Declaration of Helsinki. The study was approved by the institutional review board of the First Affiliated Hospital of Nanjing Medical University.

The CRC cell lines (HT29 and LoVo) were obtained from the type culture collection of the Chinese Academy of Sciences (Shanghai, China) and have recently been tested for mycoplasma contamination. All cells were cultured in DMEM medium supplemented with 10% foetal bovine serum (Bioind), 1% penicillin‐streptomycin (Invitrogen) and maintained at 37°C with 5% CO_2_ in a humidified atmosphere.

### 2.2 Transfection

Lentivirus constructing of METTL3 knockdown or overexpression was obtained from Obio Technology Corp. Cells were plated in six wells dishes at 50% confluence and infected with METTL3 overexpression lentivirus (termed as METTL3), a negative control (termed as NC), METTL3 knockdown lentivirus (termed as shMETTL3#1, shMETTL3#2) and a scramble control (termed as shNC) in HT29 and LoVo cells. Pools of stable transfections were generated by selection using puromycin (4 μg/mL) for 2 weeks.

CCNE1 plasmids and negative control were obtained from GeneCopoeia (GeneCopoeia). Transfections were performed using the Lipofectamine 3000 kit (Invitrogen) according to the manufacturer's instructions.

### 2.3 RNA isolation and quantitative real‐time PCR (qRT‐PCR)

Total RNAs were extracted from tissues and cell lines using Trizol reagent (Invitrogen). The total RNA was reverse‐transcribed into cDNAs using Primescript RT Reagent (TaKaRa) according to the manufacturer's protocol. PCR reactions were carried out on StepOne Plus Real‐Time PCR system (Applied Biosystems) or LightCycler 480 (Roche) with SYBR^®^ Premix Ex Taq™ Reagent (TaKaRa). Gene expression was normalized to β‐actin. The qRT‐PCR reactions were performed in triplicate. All primers used here were listed in Table 1.

**Table 1:** Information of primer sequences used in this study

Primers name	Sequence (5′‐3′)
METTL3—forward	CACAGAGTGTCGGAGGTGATTC
METTL3—reverse	CCTGTAGTACGGGTATGTTGAGC
CCNE1—forward	ACTCAACGTGCAAGCCTCG
CCNE1—reverse	GCTCAAGAAAGTGCTGATCCC
β‐actin—forward	AGCGAGCATCCCCCAAAGTT
β‐actin—reverse	GGGCACGAAGGCTCATCATT
CCNE1—forward (MeRIP)	TGCTACTTGACCTAAGGGA
CCNE1—reverse (MeRIP)	GAACACCTGCGAGGAGAG

### 2.4 Protein isolation and Western blot

Total proteins from cell lines and tissues were lysed with cold RIPA buffer (Beyotime) containing protease inhibitors (Sigma‐Aldrich). The protein concentration was measured by bicinchoninic acid (BCA) analysis (Beyotime). Total protein was separated by SDS‐PAGE gel, and transferred onto polyvinylidene difluoride membrane (Millipore). The membrane was blocked in 5% (w/v) non‐fat milk, and incubated with the primary antibodies for anti‐METTL3 (Abcam 1:1000), anti‐CCNE1 (Abcam 1:2000), anti‐GAPDH (Abcam 1:1000) at 4°C overnight, followed by incubation with horseradish peroxidase‐conjugate secondary antibody (Proteintech, 1:5000) for 1 hour at room temperature. After washes, signals were detected using a chemiluminescence system (Bio‐Rad) and analysed using ImageJ software (NIH).

### 2.5 Cell proliferation and colony formation assay

HT29 and LoVo cells were seeded on a 96‐well plate with the density of 2000/3000 cells per well. Cell proliferation was assessed using cell counting kit‐8 (Obio Technology Corp) after 1, 2, 3, 4 and 5 days. After 1 hour, the absorbance at 450 nm was read on the microplate reader at 37°C. For colony formation assay, 300 cells were seeded into 6‐well plates and maintained in DMEM medium containing 10% FBS for 10‐14 days. The colonies were fixed in 4% paraformaldehyde for 30 minutes and stained with 0.1% crystal violet. Then visible colonies were photographed and counted.

### 2.6 Cell cycle assay

HT29 and LoVo cells were harvested and fixed in ice‐cold 70% ethanol. The fixed cells were washed twice with PBS and stained with propidium iodide utilizing the cycle test plus DNA reagent kit (BD Biosciences) in accordance with the manufacturer's instructions. Then, the ratios of cells in the G1, S and G2 phase were measured by flow cytometry (CytoFLEX; Beckman).

### 2.7 Xenograft formation in vivo

The animal study was approved by the Animal Research Ethics Committee of Nanjing Medical University (acceptance no.: IACUC1804027). The 4‐ to 5‐week‐old BALB/c nude mice were obtained from Medical Laboratory Animal Center of Nanjing Medical University and maintained under specific pathogen‐free conditions. LoVo cells (5 × 10^6^ cells/ 200 μL PBS) stably transfected with METTL3 knockdown lentiviral vector or control vector were respectively injected subcutaneously into the left flank of each mouse. The tumour volumes and mouse weight were assessed every 5 days. Twenty days after injection, all mice were sacrificed and tumours were surgically removed, weighed, fixed, and embedded for haematoxylin‐eosin (H&E) and immunohistochemistry (IHC) staining.

### 2.8 Immunohistochemistry analysis

Colorectal cancer tissues and their adjacent nontumorous samples were obtained from patients of the First Affiliated Hospital of Nanjing Medical University. All specimens were wrapped in paraffin and then sectioned. The sections were subjected to heat‐induced antigen retrieval, followed by blocking with 5% BSA solution and incubated with the indicated primary antibodies overnight at 4°C. Subsequently, the sections were incubated with secondary antibody at 37°C for 1 hour and stained with diaminobenzidine and counterstained by haematoxylin. The IHC localization was scored in a semi‐quantitative fashion incorporating both the intensity and distribution of specific staining. Survival curves were generated using the Kaplan‐Meier method and compared using the log‐rank test. All patients are involved in the calculation.

### 2.9 Dot blot

Cellular total RNA was isolated using Trizol reagent. RNA concentration was measured by NanoDrop Nd‐1000 spectrophotometer (Agilent). The protocol of dot blot was referred to He's study.^53^ RNA was firstly denatured by heating at 95°C for 5 minutes and transferred onto an amersham hybond‐n+ membrane (GE Healthcare) with a Bio‐Dot apparatus (Bio‐Rad). Then, the mRNA was cross‐linked to the membrane through ultraviolet. After washed by PBST, the membrane was blocked with non‐fat milk, incubated with anti‐m6A antibody (Abcam, 1:1000) overnight at 4°C and subsequently incubated with horseradish peroxidase‐conjugate secondary antibody for 1 hour at room temperature. After washes, signals were detected using a chemiluminescence system (Bio‐Rad). The membrane stained with 0.02% methylene blue (MB) in 0.3 mol/L sodium acetate (pH 5.2), was used to ensure consistency among different groups.

### 2.10 Methylated RNA immunoprecipitation

The protocol of methylated RNA immunoprecipitation (MeRIP) was referred to Meng's study.^54^ Total RNA was extracted from stable METTL3 knockdown (shMETTL3) or METTL3 control (shNC) LoVo cells with Trizol reagent and then treated with DNase R (Qiagen). Chemically fragmented RNA (~100 nucleotides) was immunoprecipitated with anti‐m6A antibody (Abcam), washed three times with IP buffer (10 mmol/L Tris‐HCl, 150 mmol/L NaCl and 0.1% [vol/vol] Igepal CA‐630 [Sigma‐Aldrich]). RNA was eluted from the beads by incubating with elution buffer (IP buffer and 6.7 mmol/L N6‐methyladenosine salt [Sigma‐Aldrich]) for 1 hour at 4°C. Following ethanol precipitation, input and eluted RNA were then analysed by qRT‐PCR.

### 2.11 Luciferase reporter assay

Stable METTL3 knockdown (shMETTL3) or METTL3 control (shNC) HT29 and LoVo cells were co‐transfected with plasmids containing 3′‐UTR of wild or mutant fragments from CCNE1 using Lipofectamine 3000 (Invitrogen) according to the manufacturer's protocols. Luciferase activity was measured using the dual‐luciferase reporter assay system (Promega) after 48 hours of incubation. Finally, relative luciferase activity was normalized to the renilla and each assay was repeated in three independent experiments.

### 2.12 RNA stability assay

To measure the half‐life of endogenous CCNE1 mRNA, actinomycin D (Act D, 2 μg/mL; Sigma‐Aldrich) was added into the transfected HT29 or LoVo cell culture medium. Total RNAs were harvested at the times indicated and then subjected to qRT‐PCR analysis. Relative expression level of transcript was normalized to β‐actin.

### 2.13 Bioinformatic analysis

Clinical data for bioinformatic analysis were downloaded from database of The Cancer Genome Atlas (TCGA) (https://cancergenome.nih.gov/) Gene Expression across Normal and Tumor tissue (http://medical-genome.kribb.re.kr/GENT/) and Gene Expression Omnibus (GEO) (https://www.ncbi.nlm.nih.gov/geo/).

### 2.14 Butyrate treatment

To observe the effect of butyrate on CRC cells, endogenous butyrate (2 mmol/L or 4 mmol/L) was added into the CRC cell culture medium. Total RNAs or proteins were harvested at the times indicated, and then subjected to qRT‐PCR analysis or Western blot. Cell proliferation assay and colony formation were also taken after butyrate treatment.

### 2.15 Statistical analysis

All data were presented as the means ± standard deviation. The comparisons between the groups were analysed by Student's *t* test. Statistical analysis was performed using the SPSS 19.0 software (SPSS), and graphical presentations were conducted with GraphPad Prism 5 software. Pearson's correlation coefficient analysis was used to analyse the correlations. *P* < .05 was considered statistically significant. Survival package was used to analyse survival from GEO database.

## 3 RESULTS

### 3.1 METTL3 was frequent up‐regulated in CRC tissues and predicted a poor prognosis in CRC patients

**Figure 1:**
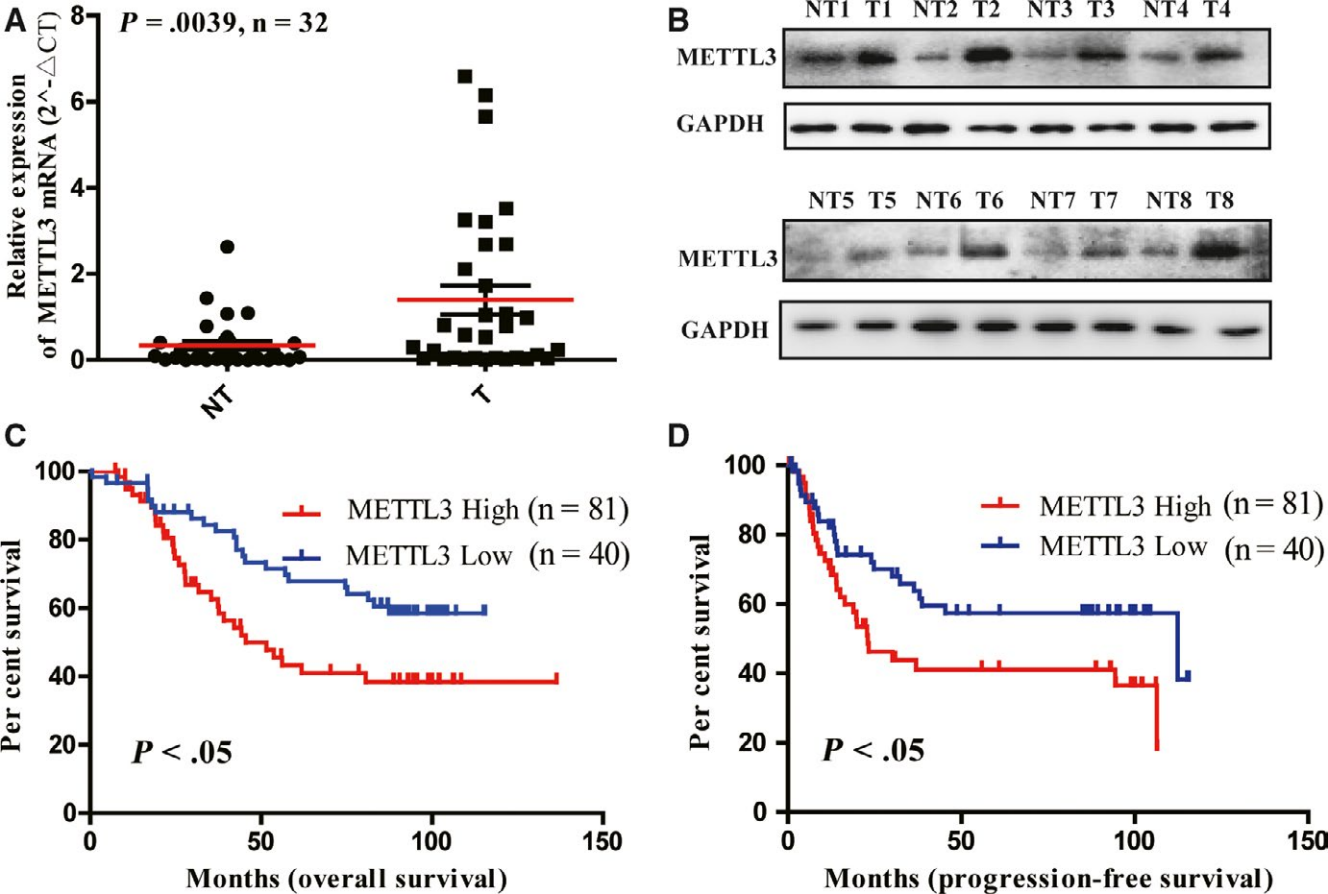
Methyltransferase like 3 (METTL3) was significantly up‐regulated in colorectal cancer (CRC) tissues and served as a prognostic factor for CRC patients. (A) Relative expression of METTL3 mRNA in 32 paired fresh CRC tissues (T) and matched adjacent normal tissues (NT) quantified by qRT‐PCR. METTL3 was expressed significantly higher in CRC tissues, compared with that in adjacent normal tissues. (B) The typical expression of METTL3 protein in 8 paired CRC tissues and adjacent normal tissues by Western blot. Kaplan‐Meier survival curves of overall survival (C) and progression‐free survival (D) in 121 CRC patients based on METTL3 expression analysed by immunohistochemistry (IHC) staining. The log‐rank test was used to compare differences between two groups (*P* < .05)

Firstly, qRT‐PCR analysis of 32 paired CRC tissues and, Western blot analysis of eight paired CRC tissues and TCGA database analysis demonstrated that METTL3 was dramatically up‐regulated in CRC tissues, compared with the paired adjacent tissues both in mRNA and protein levels (Figure 1A,B and Figure S1A). Kaplan‐Meier analysis showed that CRC patients with high METTL3 expression had both shorter overall survival (OS) and progression‐free survival (PFS), compared with those with low expression of METTL3 (Figure 1C,D). Meanwhile, GEO database analysis also showed that high METTL3 expression worsened overall prognosis in CRC patients (Figure S1B).

### 3.2 METTL3 promoted CRC cells proliferation in vitro and in vivo

**Figure 2:**
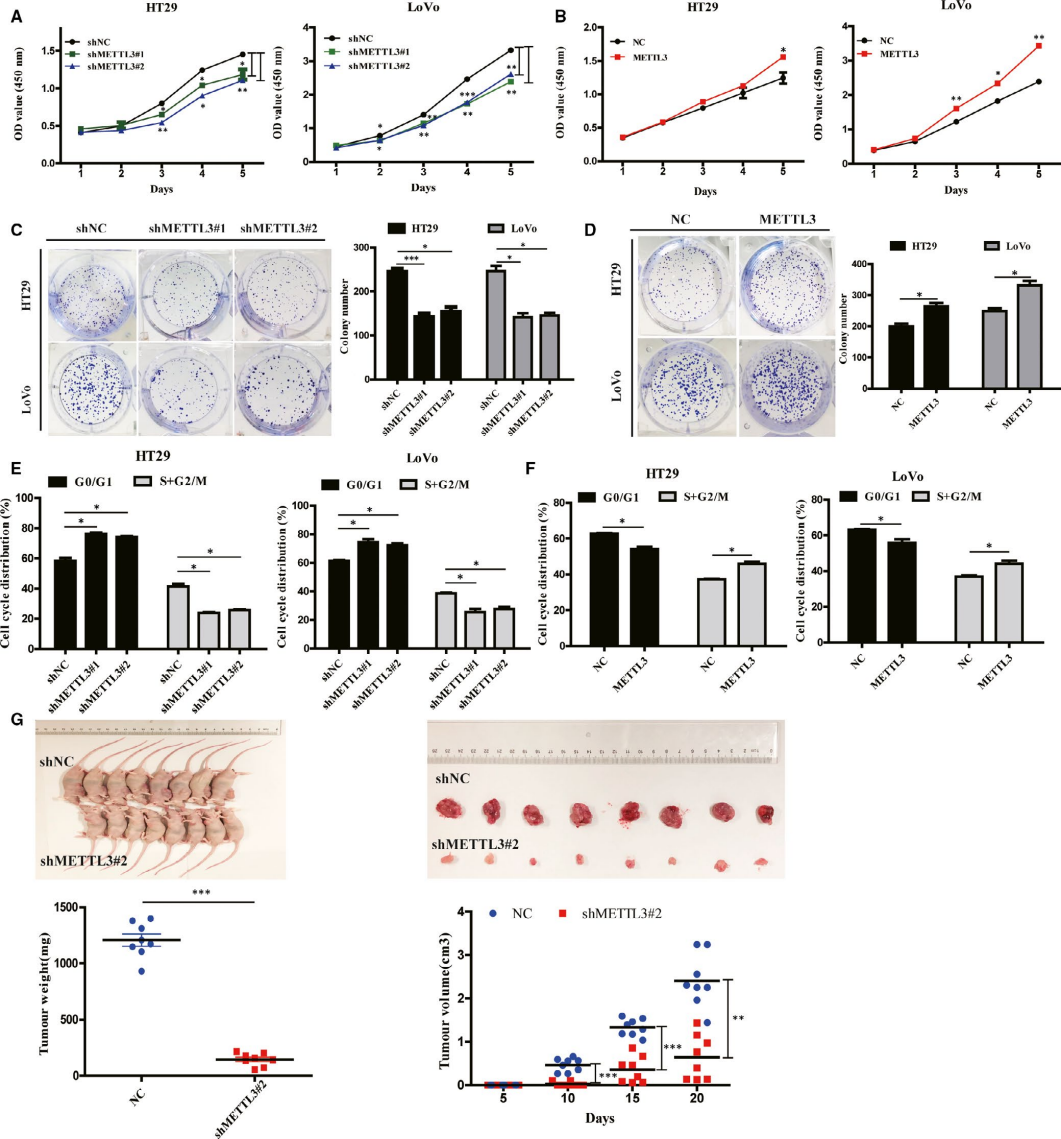
Methyltransferase like 3 (METTL3) promoted colorectal cancer (CRC) cell proliferation in vitro and in vivo. Effects of METTL3 knockdown or overexpression on cell proliferation (A and B), colony formation (C and D) and cell cycle (E and F) measured by CCK‐8 assays, colony formation assays and flow cytometry in HT29 and LoVo cells. Effects of METTL3 knockdown (shMETTL3#2) (G) on tumour volume and weight in nude mouse xenograft models (n = 8 for each group). Data represented the mean ± SD, *P < .05, **P < .01, ***P < .001

First of all, qRT‐PCR and Western blot analysis revealed that METTL3 was stably knockdown and overexpressed in HT29 and LoVo cells (Figure S2). CCK‐8 and colony formation assays indicated that METTL3 knockdown significantly inhibited CRC cells proliferation and colony formation, whereas METTL3 overexpression promoted CRC cells proliferation and colony formation (Figure 2A‐D). We can find OD values of METTL3 overexpression group were higher than that of NC group, and it was significant only in the Day 5. We thought it may be due to the oncogenic activity of METTL3 in CRC. The function of its overexpression in cells may be not as obvious as that in its knockdown. Cell cycle assays indicated that METTL3 knockdown induced G1‐S cell cycle arrest, and METTL3 overexpression decreased percentage of G1 phase (Figure 2E,F and Figure S3A,B). In subcutaneous implantation experiment, METTL3 knockdown could inhibit CRC tumour size and weight in mice (Figure 2G and Figure S4).

### 3.3 METTL3 regulated cyclin E1 expression in CRC cells and correlated with CCNE1 expression in human CRC tissues

**Figure 3:**
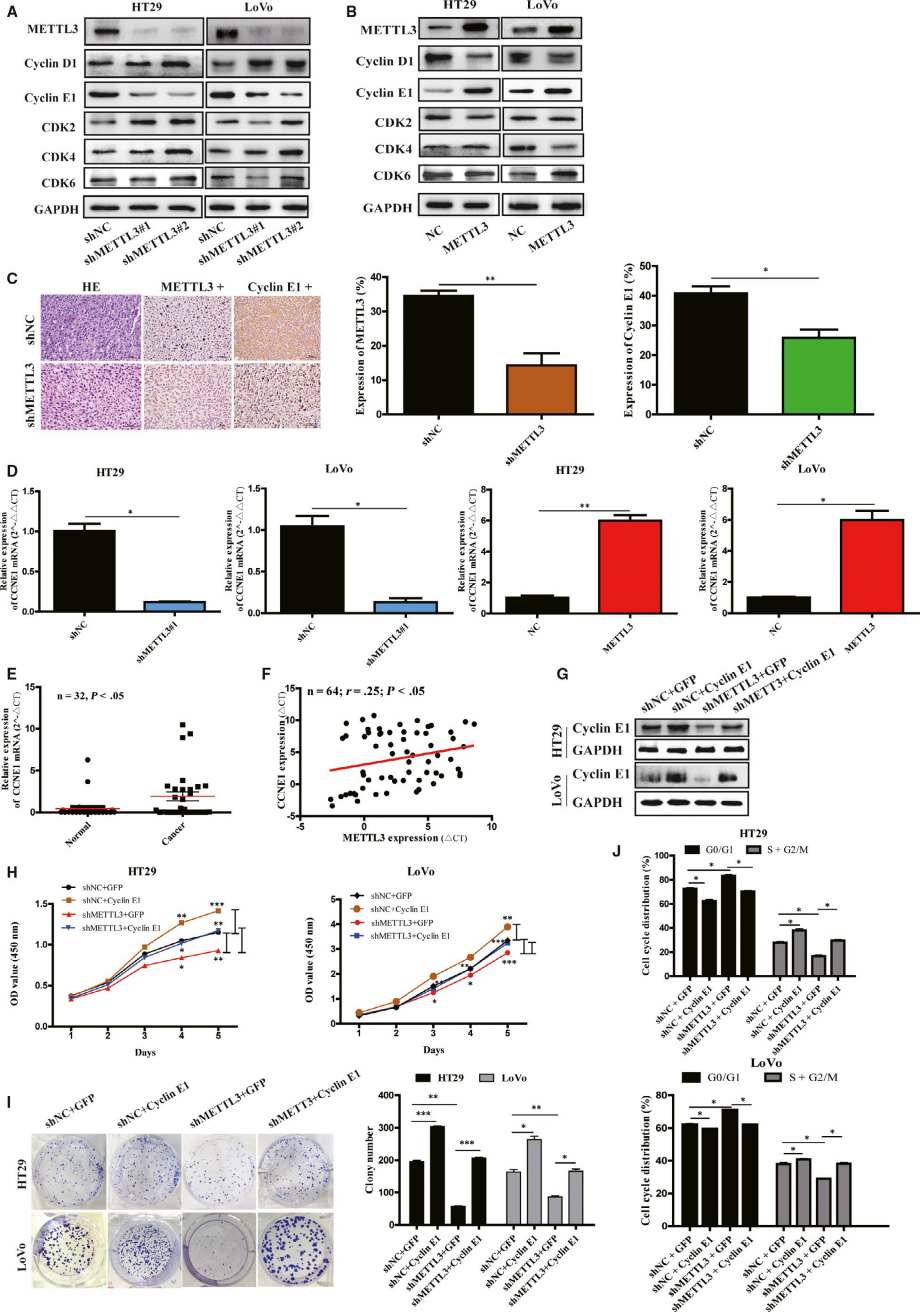
Cyclin E1 was positively correlated with methyltransferase like 3 (METTL3) expression in colorectal cancer (CRC), and overexpression of cyclin E1 reversed the cell proliferation affected by METTL3 knockdown in CRC cells. (A) The expression of cyclin D1, cyclin E1, CDK2, CDK4 and CDK6 was measured by Western blot in HT29 and LoVo cells with METTL3 knockdown or overexpression (A, B). Cyclin E1 expression decreased in METTL3 knockdown cells whereas increased in METTL3 overexpression cells, obviously. immunohistochemistry (IHC) staining and the histogram of IHC analysis showed expression of cyclin E1 significantly decreased in METTL3 knockdown CRC xenografts in nude mice (C). Relative expression of CCNE1 mRNA was measured by qRT‐PCR in HT29 and LoVo cells with METTL3 knockdown or overexpression (D). (E) CCNE1 mRNA was up‐regulated in 32 paired CRC tissues, compared with adjacent normal tissues by qRT‐PCR. (F) A moderate positive correlation between the expression of METTL3 and CCNE1 mRNA was showed in 32 paired CRC tissues as compared with adjacent normal tissues by qRT‐PCR and Pearson correlation analysis. The mRNA expression was quantified by ∆*C*_T_. (G) Western blot of cyclin E1 in HT29 and LoVo cells co‐transfected with lentivirus vector (METTL3 knockdown or control) and plasmid vector (CCNE1 or control). Overexpression of cyclin E1 rescued the cell proliferation (H), colony formation (I), cell cycle arrest (J) affected by METTL3 knockdown in HT29 and LoVo cells by CCK‐8 colony formation assays and cell cycle assay. Data represented the mean ± SD, *P < .05, **P < .01, ***P < .001

To investigate the potential mechanism of CRC cells proliferation regulated by METTL3, we used Western blot and qRT‐PCR to investigate the expression of cell cycle‐related proteins, cyclin D1, cyclin E1, CDK2, CDK4 and CDK6 by the knockdown or overexpression of METTL3. We found that knockdown of METTL3 could prominently decrease cyclin E1 and CCNE1 expression (Figure 3A,D and Figure S5C), whereas METTL3 overexpression increasing cyclin E1 and CCNE1 expression, compared with the control groups (Figure 3B,D and Figure S5C). It indicated that METTL3 could regulate cyclin E1 and CCNE1 expression in CRC cells. We also found that the expression of cyclin E1 significantly decreased in METTL3 knockdown CRC xenografts tissues in nude mice (Figure 3C). Moreover, CCNE1 expression was up‐regulated in CRC tissues (Figure 3E) and positively correlated with METTL3 expression in CRC tissues and adjacent noncancerous tissues by qRT‐PCR and IHC (Figure 3F and Figure S7). Furthermore, TCGA database analysis reached the same results that CCNE1 was positively correlated with METTL3 expression in human CRC tissues (Figure S6).

### 3.4 METTL3 exerted tumour‐promoting functions in CRC by regulating cyclin E1 expression

To assess the potential function of cyclin E1 in the tumour‐promoting functions of METTL3 in CRC, we transfected CCNE1 plasmids into HT29 and LoVo cells (Figure 3G). As shown in Figure 3H‐J, overexpression of cyclin E1 largely rescued the inhibition of CRC cell growth, colony formation and cell cycle arrest caused by METTL3 knockdown.

### 3.5 METTL3 increased the stability of CCNE1 mRNA in an m6A‐dependent manner

**Figure 4:**
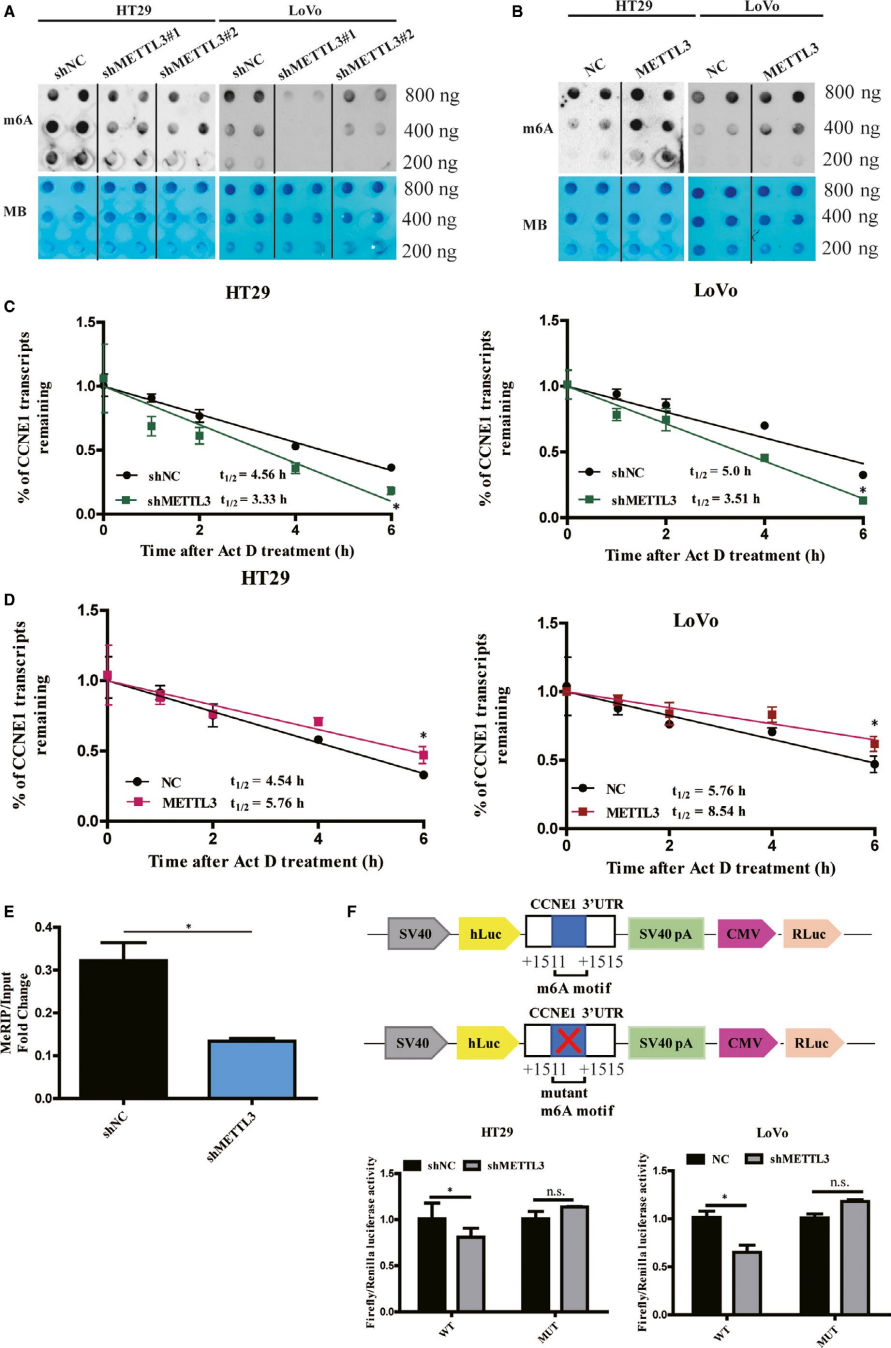
The mRNA stability of CCNE1 mRNA was regulated by methyltransferase like 3 (METTL3) in an m6A‐dependent manner. (A) m6A‐modified RNAs were measured by dot blot using m6A antibody in HT29 and LoVo cells with knockdown or overexpress of METTL3 (A and B). MB, methylene blue (as loading control). Knockdown of METTL3 shortened the half‐life of CCNE1 mRNAs (C) and overexpression of METTL3 lengthened the half‐life of CCNE1 mRNAs (D) by Act D treatment in HT29 and LoVo cells. (E) MeRIP assays showed that METTL3 knockdown caused a significant decrease in the m6A levels of the 3′‐UTR of CCNE1 mRNA in LoVo cells. Relative luciferase activity of the reporter carrying CCNE1 3′‐UTR (F) with either wild‐type or m6A sites deletion mutation in HT29 and LoVo cells. Relative luciferase activity was measured and normalized to renilla luciferase activity. Data represented the mean ± SD, *P < .05, **P < .01

Dot blot assays showed that METTL3 knockdown resulted in decreased m6A levels and overexpression of METTL3 increased m6A levels in HT29 and LoVo cells (Figure 4A,B). Knockdown and overexpression of METTL3 could shorten and prolong the half‐life of CCNE1 mRNAs, respectively (Figure 4C,D). Previous MeRIP‐Seq from Lin's study identified that the m6A level in CCNE1 mRNA could be affected by METTL3.^55^ Our further MeRIP assays confirmed that METTL3 knockdown caused a significant decrease in the m6A levels of CCNE1 mRNA in LoVo cells (Figure 4E). It was reported m6A sites existed mostly in the 3′‐UTRs of mRNAs,^56^ which can affect mRNA stability.^57^ We also found a METTL3 catalysing motif site (GGACU) in the 3′‐UTR of CCNE1 mRNA. So, we further conducted dual‐luciferase assays to elucidate whether this m6A site was required for the CCNE1 mRNA stability. We constructed the m6A mutant forms of CCNE1 where the adenosine bases in m6A consensus sequences (GGACU) were deleted, and thus abolishing m6A modification. We found the luciferase activity increased in mutant CCNE1 reporter, compared with in CCNE1‐fused reporter in METTL3 knockdown HT29 and LoVo cells (Figure 4F), which suggested METTL3 regulated cyclin E1 expression by methylating the m6A site in 3′‐UTR of CCNE1 mRNA.

### 3.6 Intestinal flora metabolite down‐regulated METTL3 expression in CRC cells

**Figure 5:**
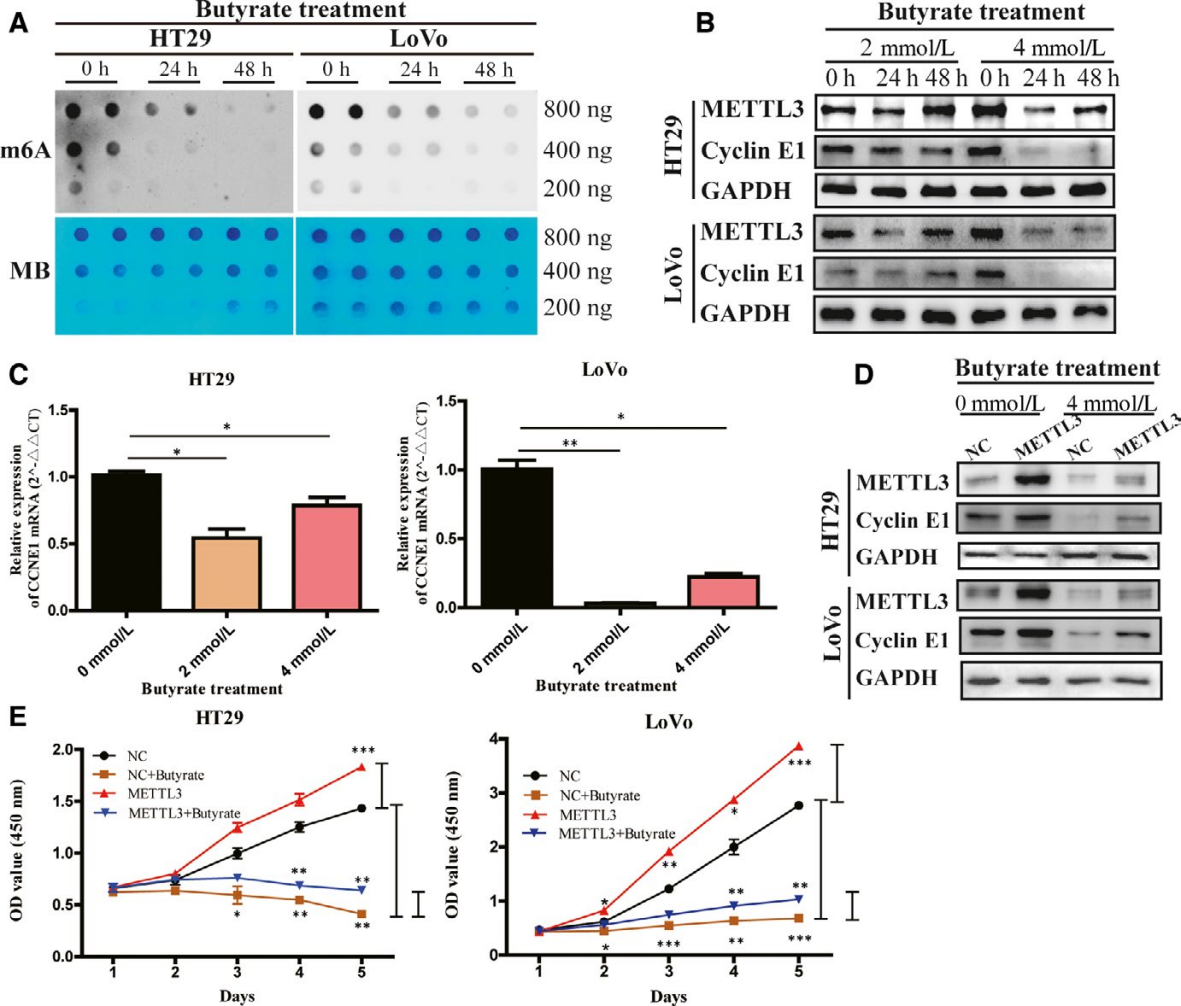
Butyrate down‐regulated methyltransferase like 3 (METTL3) expression and m6A level in colorectal cancer (CRC) cells. (A) m6A‐modified RNAs were measured by dot blot using m6A antibody in HT29 and LoVo cells with after butyrate treatment for 24/48 h. MB, methylene blue (as loading control). Expression of METTL3 and cyclin E1 in HT29 and LoVo cells after butyrate treatment was measured by Western blot. Butyrate (4 mmol/L) could down‐regulate METTL3 and cyclin E1 expression in CRC cells (B). Relative expression of CCNE1 (C) mRNA was measured by qRT‐PCR in HT29 and LoVo cells with butyrate (4 mmol/L) treatment. (D) Western blot of METTL3 and cyclin E1 in HT29 and LoVo cells co‐transfected with lentivirus vector (METTL3 overexpression or control) and then treatment of butyrate (4 mmol/L). Overexpression of METTL3 rescued the cell proliferation (E) affected by butyrate treatment in HT29 and LoVo cells by CCK‐8 assays. Data represented the mean ± SD, *P < .05, **P < .01, ***P < .001

Dot blot demonstrated that level of m6A of HT29 and LoVo cells decreased after being exposed to butyrate (4 mmol/L) for 24/48 hours (Figure 5A). To explore whether METTL3 expression is regulated by intestinal flora metabolite, we treated LoVo cells with DCA, ursodeoxycholic acid and butyrate for 24/48 hours, respectively. The result showed that butyrate (4 mmol/L) could decrease METTL3 expression in LoVo cells significantly (Figure S8A). The similar effect of butyrate was also observed in HT29 cells (Figure 5B). Moreover, METTL3 and CCNE1 mRNAs were both decreased in HT29 and LoVo cells significantly, when treated by butyrate (4 mmol/L) for 48 hours (Figure 5C and Figure S8B). In addition, METTL3 overexpression could reverse the inhibition of CRC cell growth and colony formation caused by butyrate treatment (Figure 5D,E and Figure S8C).

## 4 DISCUSSION

In this study, we gradually confirmed that METTL3, a critical m6A methyltransferase, played an oncogenic role in CRC.^37^ Moreover, our study and GEO database predicted that up‐regulated expression of METTL3 in CRC tissues was positively associated with overall survival and PFS in patients, which brought light to its potential prognostic value for CRC. In vitro, we observed that knockdown of METTL3 dramatically inhibited CRC cell proliferation and induced G1 cell cycle arrest; the opposite was true when the expression of METTL3 was overexpressed. Meanwhile, in vivo experiments confirmed the oncogenic function of METTL3 in CRC. All these results strongly supported that METTL3 played as an oncogenic role in CRC.

We found that METTL3 could significantly promote CRC cells proliferation, colony formation and cell cycle process. Therefore, we suspected that METTL3 could promote CRC proliferation by regulating CRC cell cycles. Then, we investigated the expression of cell cycle‐related proteins, cyclin D1, cyclin E1, CDK2, CDK4 and CDK6 in METTL3 knockdown or overexpression cells, and found that METTL3 showed positive relationship with cyclin E1 expression. Similar result was also found by IHC analysis of xenograft tissues. Knockdown of METTL3 could down‐regulate CCNE1 expression in CRC cells by decreasing CCNE1 mRNA stability, which was confirmed by Act D treatment. Accordingly, overexpression of CCNE1 reversed the cell proliferation inhibiting activity affected by METTL3 knockdown in CRC cells. Therefore, we identified cyclin E1 as the downstream target of METTL3 in promoting CRC proliferation. One of the main characteristics of malignant tumours is abnormal cell growth. As a regulator cyclin of the G1‐to‐S phase transition of the cell cycles, cyclin E1 has been extensively studied in cancers.^58^ Impaired expression of cyclin E1 proteins or CCNE1 mRNAs has been identified in cancers, such as colorectal adenomas and adenocarcinomas,^59^ laryngeal squamous cell carcinomas,^60^ and has been evaluated as a prognostic marker.^59^, ^60^, ^61^ Cyclin E1 overexpression increases cell proliferation by promoting progression to S phase and may lead to carcinogenesis.^62^ It leads to tumorigenesis likely due to genetic instability.^63^ In CRC, high expression of cyclin E1 in tumour increased the risk of tumour recurrence and was correlated with its malignancy, representing a possible prognostic marker.^64^, ^65^

Dot blot showed that METTL3 could increase m6A modification in CRC cells. MeRIP and dual‐luciferase assays showed that METTL3 could specifically catalyse m6A sites of 3′‐UTR in CCNE1 mRNA, which increased the stability of CCNE1 mRNAs and eventually lead to increased cyclin E1 expression. They all indicated that the oncogenic function of METTL3 is dependent on its m6A ‘writer’ activity by mainly stabilizing CCNE1 mRNAs in CRC.

Human intestinal microbiota can promote or suppress CRC, not only due to the carcinogenic activities of pathogenic bacterium but also caused by the complex effect of wider microbial community, particularly their metabolites.^39^ Recent studies demonstrated that microbiomes had strong effects on m6A modification and expression of m6A ‘writers’ in mouse brain and intestine.^40^ By consulting the literature, we found three intestinal flora metabolites that are most closely related to the development of CRC: Short‐chain fatty acids (butyrate),^66^ DCA^67^ and ursodeoxycholic acid.^68^, ^69^ In the present study, we found that butyrate, a classical intestinal microbial metabolite, could decrease the level of m6A and down‐regulate the expression of METTL3 and CCNE1 in CRC cells. In addition, METTL3 overexpression could reverse the inhibition of CRC cell growth and colony formation caused by butyrate treatment. Combined with the result that METTL3 could stabilize CCNE1 mRNAs in CRC cells, we suspected that butyrate may down‐regulate CCNE1 expression in an m6A‐METTL3‐dependent manner. Butyrate is the main source of energy for intestinal epithelial cells, which lays the foundation for a modern symbiosis theory between the gut microbiota and intestinal epithelial cells.^48^ It can inhibit the development of CRC and promote intestinal health through various mechanisms.^70^, ^71^ Here, we found the effects on m6A modification and expression of m6A ‘writers’ METTL3 may be involved in CRC cell proliferation affected by butyrate. Though the underlying mechanisms of butyrate's anticarcinogenic function have not yet been elucidated, it could promote histones acetylation^50^, ^72^and the accessibility of transcription factors to nucleosomal DNA.^45^, ^73^, ^74^ Interestingly, cancer cells appear to be more sensitive to the actions of HDACi than nontransformed cells, but the mechanistic basis for this apparent selectivity is poorly understood.^75^ Butyrate participates in the m6A modification and METTL3 expression may be conducted by one or more pathway mentioned above. It would be investigated in the further study.

**Figure 6:**
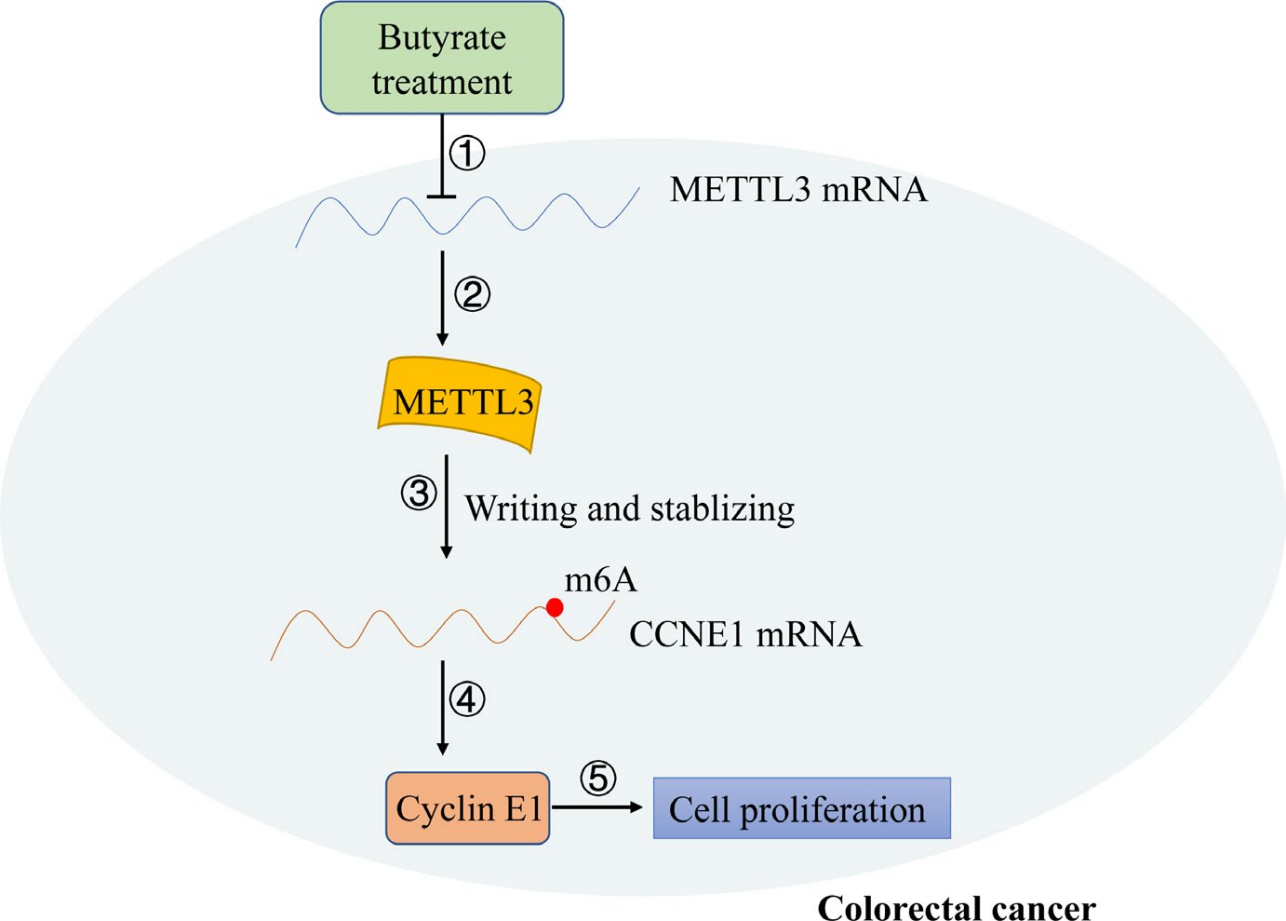
Working model: methyltransferase like 3 (METTL3) promoted colorectal cancer (CRC) cell proliferation by stabilizing CCNE1 mRNAs in an m6A‐dependent manner. ①, ②: Butyrate down‐regulated the expression of METTL3 mRNA and protein. ③: METTL3 catalysed m6A decoration in 3′‐UTR of CCNE1 mRNAs and stabilized them. ④: METTL3 increased the level of cyclin E1 proteins. ⑤: Overexpression of cyclin E1 protein promoted cell proliferation in CRC cells

In summary, our work confirmed Li's study that METTL3, a critical m6A methyltransferase, could facilitates CRC progression, and further revealed a new mechanism that METTL3 could promote CRC cells proliferation by directly stabilizing CCNE1 mRNA in m6A‐dependent manner, representing a promising therapeutic strategy for the treatment of CRC. Moreover, the effects on m6A modification and expression of METTL3 may be involved in CRC cell proliferation affected by butyrate (Figure 6).

## CONFLICTS OF INTEREST

The authors confirm that there are no conflicts of interest.

## AUTHORS’ CONTRIBUTION

Wei Zhu, Ji‐Fu Wei, Qiang Ding and Lingjun Zhu conceptualized and designed the study. Wei Zhu, Yan Si and Jing‐Zi Wang developed the methodology. Wei Zhu, Yu Lin, Mengda Cao and Shanwen Sun analysed and interpreted the data. Wei Zhu, Yan Si and Ji‐Fu Wei wrote, reviewed and revised the manuscript. Wei Zhu, Yan Si and Jun Xu involved in acquisition of data and gave administrative, technical or material support. Ji‐Fu Wei supervised the study.

## Data Availability

The data sets used and analysed in the current study are available from the corresponding author in response to reasonable requests.
