# Supplementary material for: Methyltransferase like 3 promotes colorectal cancer proliferation by stabilizing CCNE1 mRNA in an m6A‐dependent manner
Source: J Cell Mol Med. 2020 Feb 10;24(6):3521–33. doi: 10.1111/jcmm.15042 (PMC7131945; doi:10.1111/jcmm.15042)

**Figure S1:** METTL3 was upregulated in CRC tissues by TCGA database analysis (Figure 1A), which predicted a poor prognosis in CRC patients by GEO database analysis (Figure 1B).


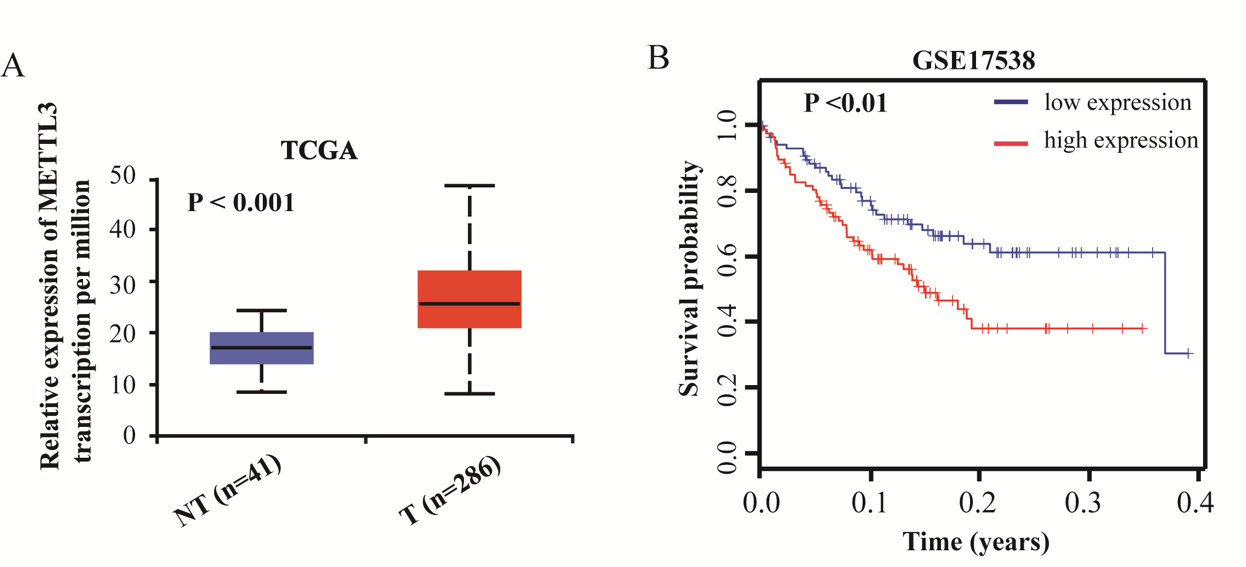


**Figure S2:** The efficiency of METTL3 knockdown or overexpression in HT29 and LoVo cell lines was measured by qRT-PCR (A and B) and Western blot (C and D).


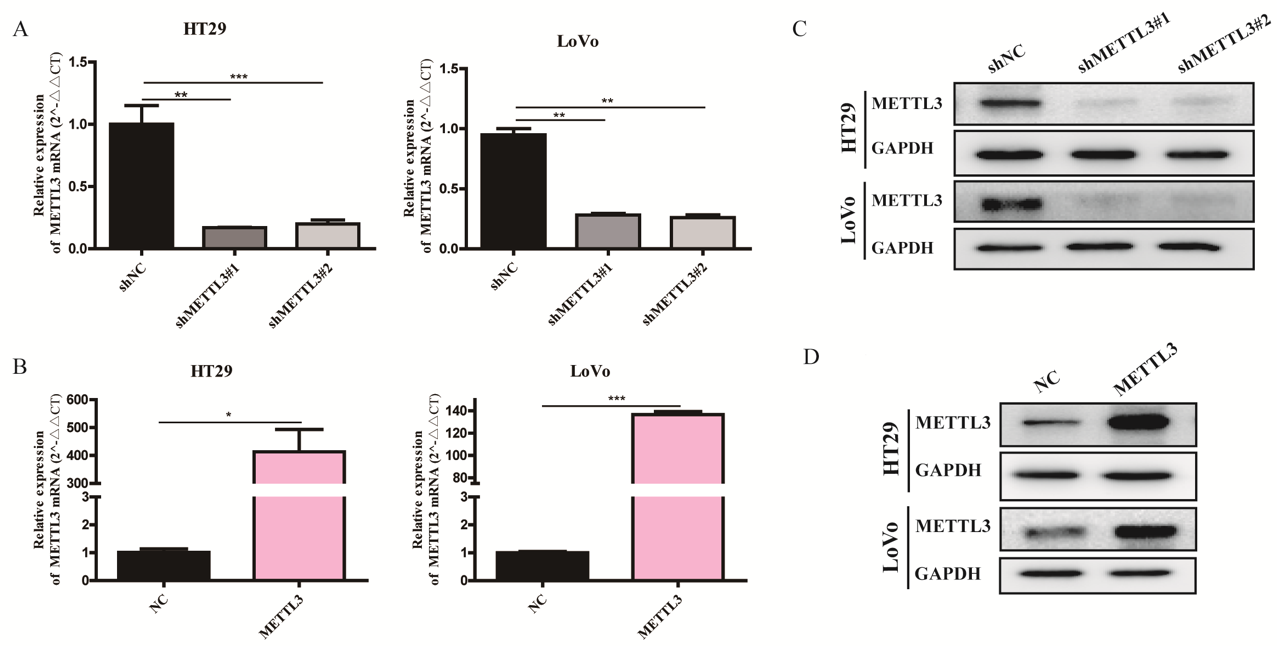


**Figure S3:** Effects of METTL3 knockdown or overexpression on cell cycles (A, B) in HT29 and LoVo cells.


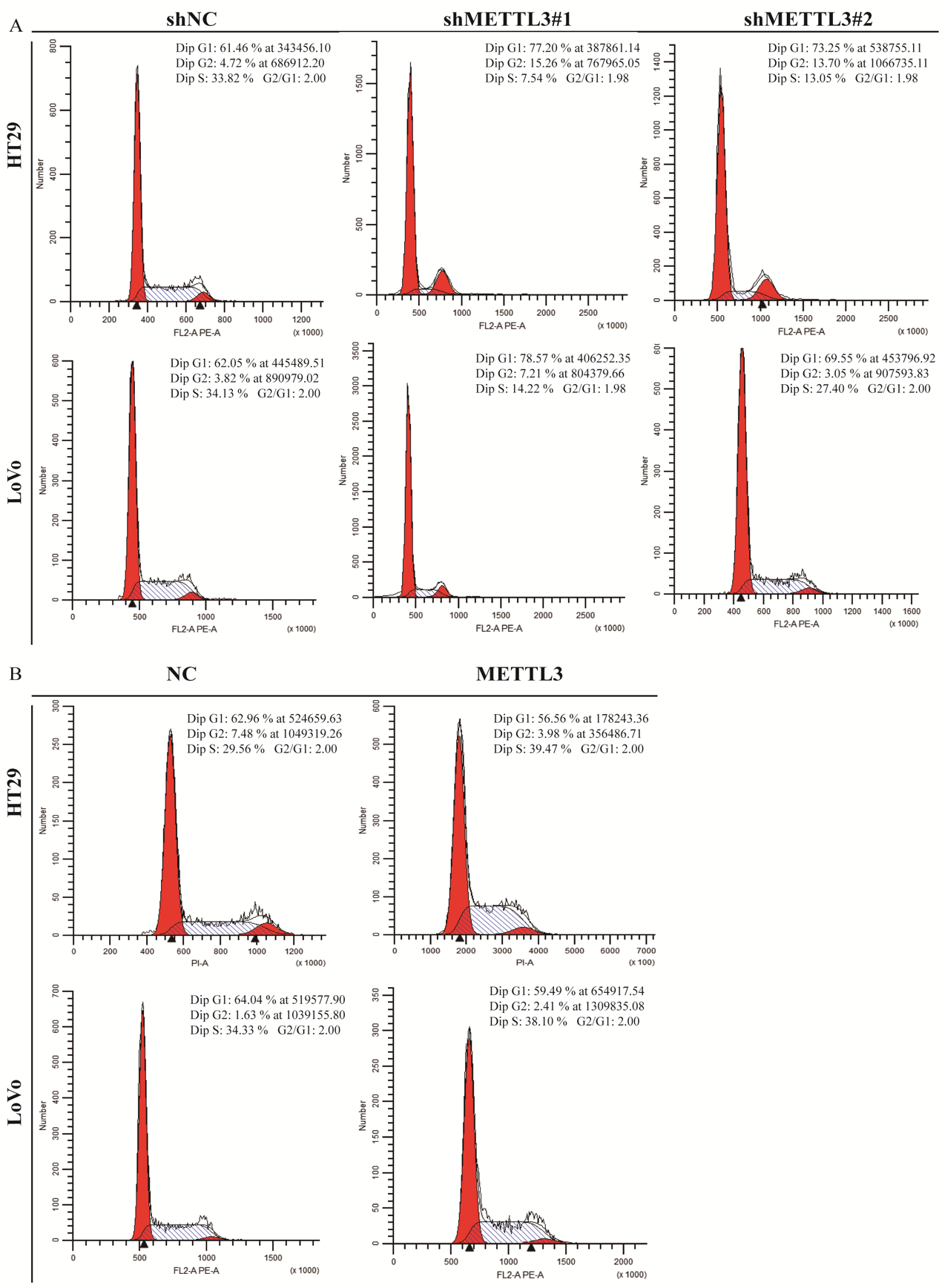


**Figure S4:** Effects of METTL3 knockdown (shMETTL3#1) on tumor volume and weight in nude mouse xenograft models (n=5 for each group). Data represented the mean ± SD, * P < 0.05, ** P < 0.01, *** P < 0.001.


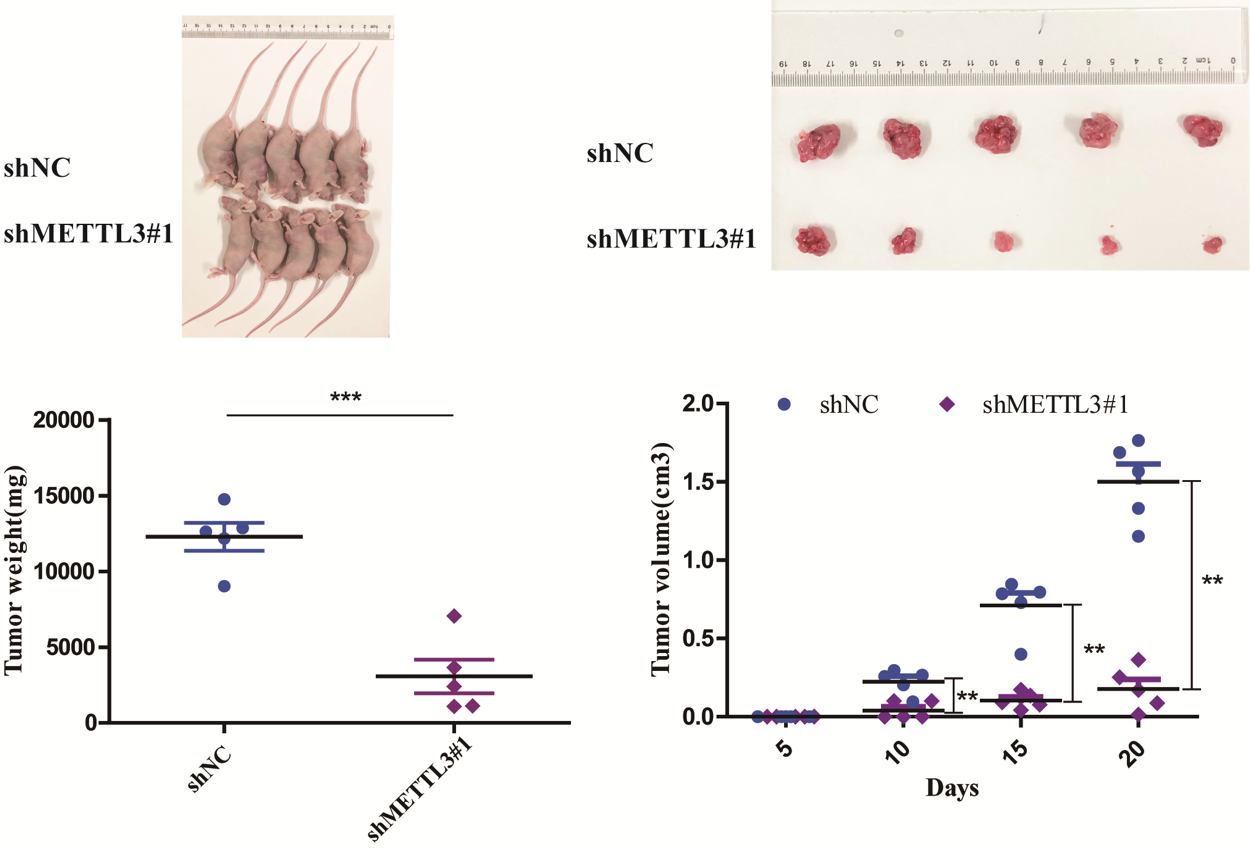


**Figure S5:** **CCNE1 mRNA expression was positively correlated with METTL3 mRNA expression in CRC.** The mRNA expression of METTL3 (A), CCND1 (B), CCNE1 (C), CDK2 (D), CDK4 (E) and CDK6 (F) were measured by qRT-PCR in HT29 and LoVo cells with METTL3 knockdown or overexpression. Data represented the mean ± SD, ***** P < 0.05, ****** P < 0.01.


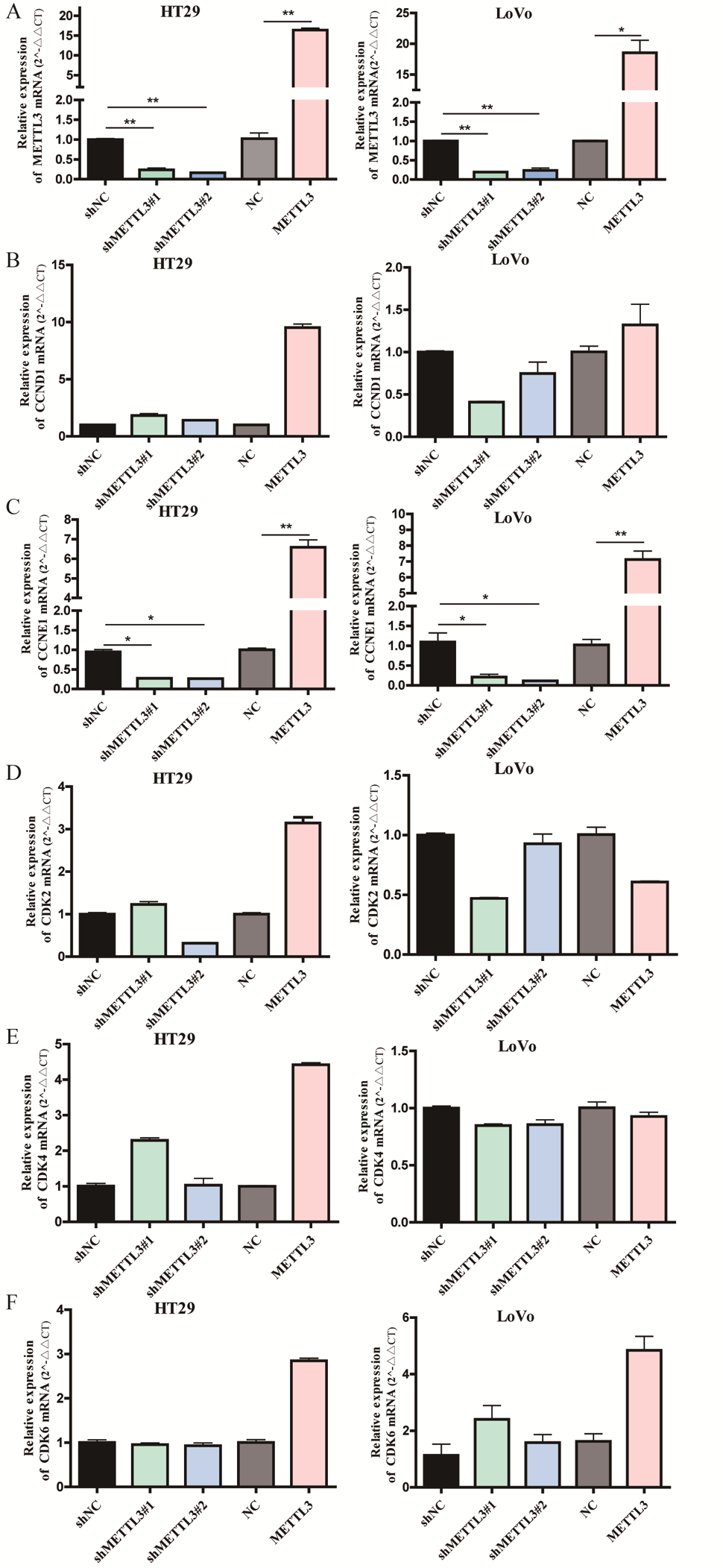


**Figure S6:** TCGA database demonstrated the positive correlation between CCNE1 and METTL3 mRNA in CRC patient tissues.


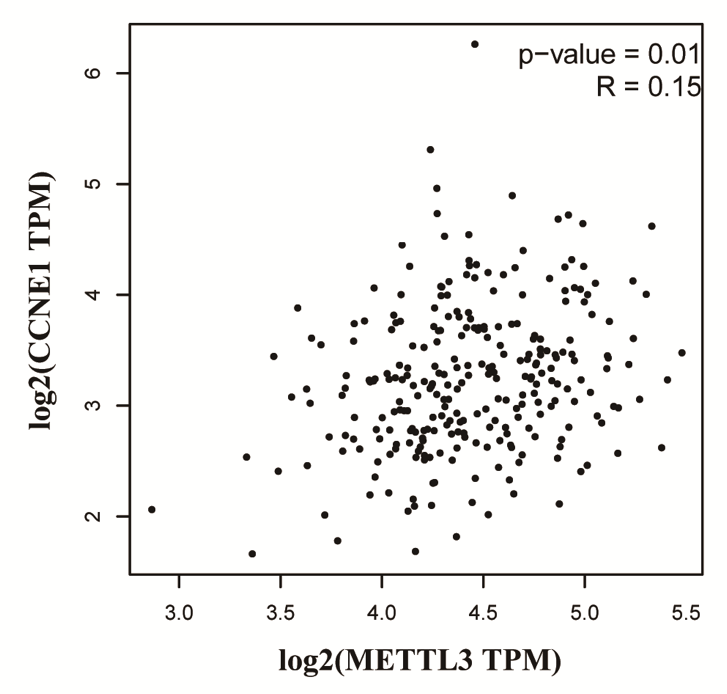


Figure S7: IHC analysis of METTL3 and Cyclin E1 in 5 paired CRC tissues (T) as compared with adjacent normal tissues (NT). Data represented the mean ± SD, * P < 0.05.
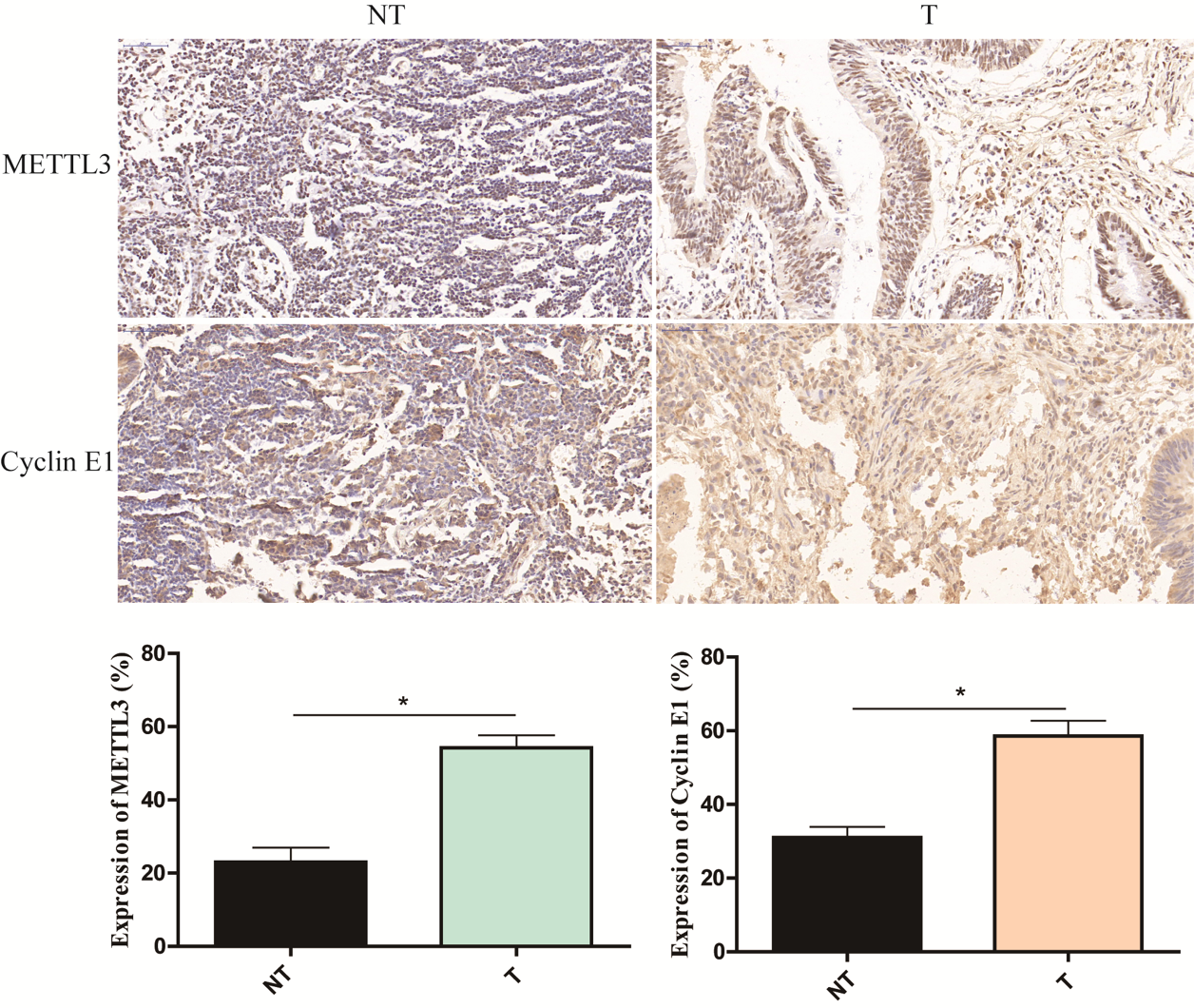


**Figure S8:** (A) Expression of METTL3 in LoVo cell after intestinal flora metabolite treatment was measured by Western blot. Butyrate (4 mM) can downregulated METTL3 expression in LoVo cell. Relative expression of METTL3 (B) mRNAs were measured by qRT-PCR in HT29 and LoVo cells with butyrate (4 mM) treatment. Overexpression of METTL3 rescued the colony formation (C) affected by butyrate treatment in HT29 and LoVo cells by colony formation assays.


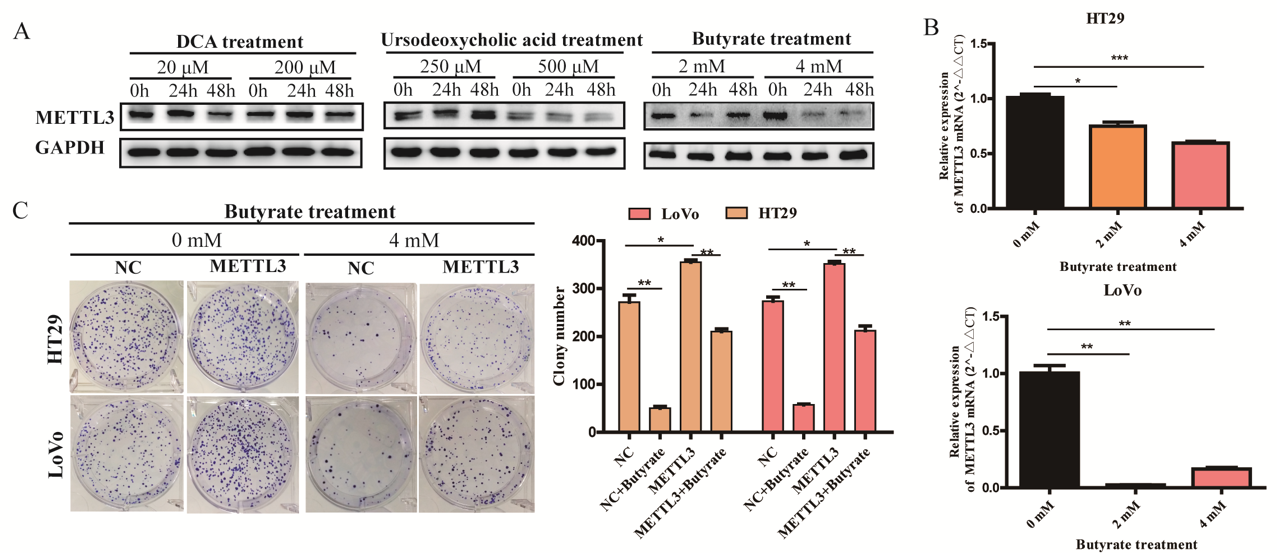

Supplement: Supplementary file 1 [file JCMM-24-3521-s001.docx]
